# Suppression of MD2 inhibits breast cancer in vitro and in vivo

**DOI:** 10.1007/s12094-021-02587-9

**Published:** 2021-03-17

**Authors:** S. Zheng, W. Fu, R. Ma, Q. Huang, J. Gu, J. Zhou, K. Lu, G. Guo

**Affiliations:** 1 Department of Breast Surgery, The First Affiliated Hospital of Wenzhou Medical University, Ouhai District, Wenzhou, 325000 Zhejiang China; 2 Department of Breast Surgery, The Second Affiliated Hospital of Wenzhou Medical University, Lucheng District, Wenzhou, 325000 Zhejiang China

**Keywords:** Breast neoplasms, Myeloid differentiation 2 (MD2), Proliferation, Migration, Invasion

## Abstract

**Purpose:**

To explore the effects of the intervening measure targeting myeloid differentiation 2 (MD2) on breast cancer progression in vitro and in vivo.

**Methods:**

The expression of MD2 in normal breast cells (Hs 578Bst) and three kinds of breast carcinoma cell lines (MCF-7, MDA-MB-231 s and 4T1) were detected by western blot. MTT assay was used to detect the proliferation of 4T1 cells treated by L6H21, cell migration and invasion was measured by wound healing assay and trans-well matrigel invasion assay, respectively. In addition, to further study the role of MD2 in tumor progression, we assessed the effects of inhibition of MD2 on the progression of xenograft tumors in vivo.

**Results:**

The expression of MD2 is much higher in MDA-MB-231 s and 4T1cells than that in normal breast cells (Hs 578Bst) or MCF-7 cells (*p* < 0.05). In vitro, suppression of MD2 by L6H21 has a significant inhibition of proliferation, migration and invasion in 4T1 cells in dose-dependent manner. In vivo, L6H21 pretreatment significantly improved survival of 4T1-bearing mice (*p* < 0.05). Additionally, we also observed that none of the mice died from the toxic effect of 10 mg kg^−1^ L6H21 in 60 days.

**Conclusion:**

Overall, this work indicates that suppression of MD2 shows progression inhibition in vitro and significantly prolong survival in vivo. These findings provide the potential experimental evidence for using MD2 as a therapeutic target of breast carcinoma.

## Introduction

Breast cancer is one of the most common cancers affecting women worldwide [1]. Despite intensive efforts and remarkable advances in the management of breast cancer, the physiological conditions that lead to tumorigenesis including breast cancer are not well understood and distant metastasis are still occurred in part of patients after treatments. It was reported that over 90% of the deaths of cancer patients are caused by metastasis [2]. Therefore, finding new modalities that treat the local and systemic components of the disease has become increasingly important.

Toll like receptors (TLRs) belong to the pathogen recognition receptors (PRRs) family, which are essential components of innate immune system and serve as major contributor to chronic inflammation [3]. Through identifying pathogen-related molecular patterns (PAMPs) and damage-related molecular patterns (DAMPs), TLRs plays a central role in the immune response [4]. There are two pathways that TLRs use to send their messages to regulate cell functions. TLR3 uses TIR domain-containing adapter-inducing interferon-β (TRIF), while TLR1, TLR2, TLR5, TLR6, TLR7, TLR8 and TLR9 use myeloid differentiation primary response (MYD88). TLR4, however, uses both MYD88 and TRIF pathways for transduction of its signaling [5]. TLRs binding to ligands can activate a variety of cascade signal transduction pathways and promote the release of inflammatory mediators, such as cytokines, chemokines, and ultimately promote tumor cell growth, angiogenesis and lymphangiogenesis, tumor infiltration and metastasis [6, 7]. As one of the most unique TLRs, TLR4 has already been linked to tumors including breast cancer and has been implicated in low overall survival rate [8–11].

MD2, well known as an accessory protein of TLR4, plays an essential role in activation of TLR4 signaling pathway in inflammatory response [12]. However, whether MD2 has a similar effect on the progression of breast cancer is still poorly understood and there was rare evidence indicating the correlation of MD2 and tumor progression. Previously, our cooperators synthesized a new chalcone derivative, (E)-2, 3-dimethoxy-4′-methoxychalcone (L6H21), which identified MD2 as its molecular target. In addition, they demonstrated that L6H21 shows excellent inhibition of the TLR4-mediated inflammatory response and septic injury both in vitro and in vivo [13]. Therefore, to investigate whether L6H21 has antitumor effects, in this study, we first detected the expression of MD2 in four kinds of cell lines by western blot assay. Next, we aimed to observe and confirm that the effects of L6H21 on 4T1 cells proliferation, migration and invasion in vitro. Additionally, model of transplanted tumor on BALB/c nude mouse were used to study the anticancer effect of L6H21 in vivo*.*

## Materials and methods

### Cell culture

Normal breast cells (Hs 578Bst) and breast cancer cell lines MCF-7 (estrogen receptor-positive, HER2-negative breast cancer), MDA-MB-231 s (the estrogen receptor-negative, progesterone receptor-negative and HER2-negative human breast cancer cells) and 4T1 (spontaneously metastasizing mammary adenocarcinoma) were obtained from ATCC and grown according to ATCC recommended culture conditions. MDA-MB-231 s and 4T1 cells represent highly malignant breast cancers. The cells were cultured in DMEM (Gibco) or RPMI 1640 medium (Gibco) with 10% fetal bovine serum (FBS) (Gibco), and then incubated at 37 °C in a humidified atmosphere containing 5% CO_2_ and 95% air.

### Western blot

Cell protein samples (50 μg) were subjected to 10% SDS-PAGE and transferred onto a PVDF membrane (Bio-Rad Laboratories). After being blocked in blocking buffer (5% milk in tris-buffered saline containing 0.05% Tween 20) for 1.5 h at room temperature, membranes were incubated with different primary antibodies overnight at 4 °C. The membranes then were washed in TBS-T and reacted with secondary horseradish peroxidase-conjugated antibody (Santa Cruz, CA, USA;1:5000) for 1–2 h at room temperature. Blots were then visualized using enhanced chemiluminescence reagents (Bio-Rad Laboratories). The density of the immunoreactive bands was analysed using Image J software (NIH, Bethesda, MD, USA).

### MTT assay

Cell proliferation was determined using the MTT method. Briefly, 48 h after treatment, the MCF-7, MDA-MB-231 s and 4T1 cells were seeded into 96-well culture plates (BD Biosciences, Franklin Lakes, NJ, USA) and incubated overnight at 37 °C in 5% CO_2_. Cell proliferation was assessed at 24, 48, 72, 96 and 120 h following addition of 0.5 mg ml^−1^ MTT (Sigma, USA) solution. After a 4-h incubation, the reaction was stopped by addition of 150 µl DMSO (Sigma). After 10 min of agitation (100 rpm), the optical density (OD) at 490 nm was determined with microplate reader (BioTek). Each sample was tested with six replicates. All experiments were performed in biological triplicate.

### Wound migration assay

The 4T1 cells were grown in six-well plates to a confluent monolayer and subsequently wounded with sterile pipette tips. The wounded monolayers were then incubated with lipopolysaccharide (LPS) (Sigma-Aldrich) and corresponding protein as indicated in Fig. 3a for 48 h. The wound area was measured under microscope. The percentage of wound healing rate was estimated as follows: Wound healing rate % = [1 − (wound width at 48 h/wound width at 0 h)] × 100%. 100 × microscopic fields under microscope.

### Trans-well Matrigel invasion assay

Tumor cell invasion was performed using Trans-well system (Millipore) with 8 µm-pore polycarbonate filter membrane. The upper chamber was covered with Matrigel (Sigma-Aldrich) and incubated at 4 °C overnight. The upper chamber was then seeded with 1 × 10^4^ 4T1 cells incubated with LPS and corresponding protein as indicated in Fig. 3b and inserted into the lower chamber containing complete medium. After incubation at 37 °C in 5% CO_2_ for 24 h, the cells on the interior of upper chamber were removed, and the polycarbonate membranes were stained with 0.1% crystal violet (BASO) for 10 min. The number of migrating cells was counted in eight randomly selected 400 × microscopic fields under microscope.

### Xenograft assays in nude mice

To evaluate in vivo tumorigenesis, breast carcinoma xenografting mouse model was used. Male BALB/c mice weighing 18–22 g were obtained from the Wenzhou Medical University Animal Centre and prepared for tumor implantation. All experimental procedures involving animals were performed in accordance with animal protocols approved by the Institutional Animal Use and Care Committee of Wenzhou Medical University and performed according to the institutional ethical guidelines for animal experiment. The mice were randomly divided into four groups (*n* = 8 per group). 4T1-bearing mice were injected with 3 × 10^5^ 4T1 cells (i.v. through the tail vein) 3 days after being treated with L6H21 (at 10 mg kg^−1^ or 5 mg kg^−1^) and saline by intragastric administration, respectively. To investigate the toxicity of L6H21, mice of the fourth group were only treated with L6H21 at 10 mg kg^−1^ but without inoculation of 4T1 cells. Each group of mice were then intragastrically administrated per day until death or day 60. The survival curve was made to analyse the survival rate. When the mice were dead or at day 60, the body weight data were collected.

### Statistical analysis

For all data, statistical analysis was performed in SPSS 22.0 for Windows (SPSS Inc.). All data are shown as means ± SDs. The statistical significance between groups was obtained by Student’s *t* test or one-way ANOVA test and the significance level was set at *p* < 0.05.

## Results

### MD2 expression in four kinds of cell lines

**Fig. 1:**
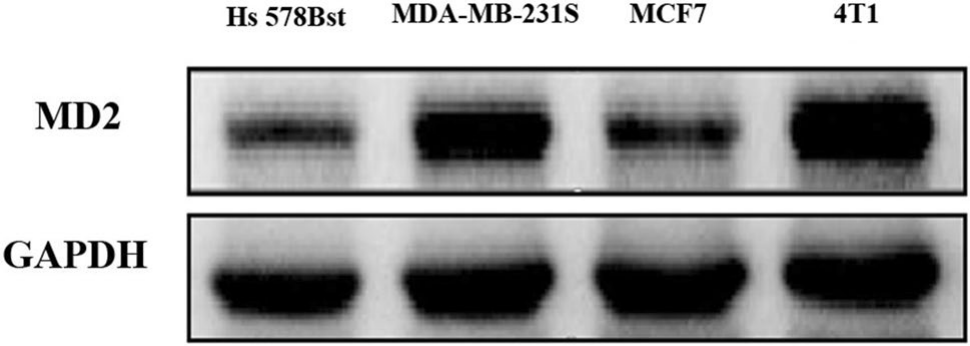
MD2 is highly expressed in MDA-MB-231 s and 4T1 cells (*p* < 0.05 compared to that expressed in Hs 578Bst cells and MCF-7 cells)

In our experiment, we first used western blot assay to examined the expression of MD2 in normal breast cells (Hs 578Bst) and three kinds of breast carcinoma cell lines (MCF-7, MDA-MB-231 s and 4T1). These cells were lysed in logarithmic phase and the total protein was extracted. MD2 protein expression was then detected by western blot. As shown in (Fig. 1), MD2 expressed in each kinds of cell lines, of these, MD2 is highly expressed in highly malignant MDA-MB-231 s and 4T1 cells.

### L6H21 inhibition of breast cancer cells proliferation

**Fig. 2:**
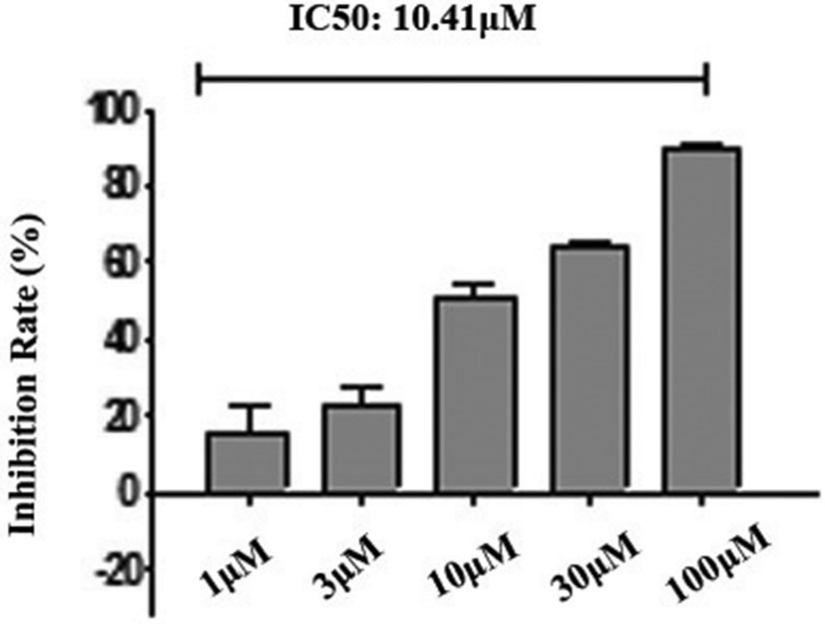
MTT assay showed that L6H21 (10 μM, 30 μM and 100 μM) significantly inhibited the proliferation of 4T1 cells in dose-dependent manner

To explore the role of MD2 in breast cancer cells proliferation, we assessed the effects of inhibition of MD2 by L6H21 in 4T1 cells by MTT assay. As shown in (Fig. 2), L6H21 significantly inhibited the proliferation of 4T1 cells in dose-dependent manner (IC_50_ = 10.41 μM), which has a similar inhibition effect in each group by curcumin, respectively.

### L6H21 inhibition of breast cancer cells migration and invasion

**Fig. 3:**
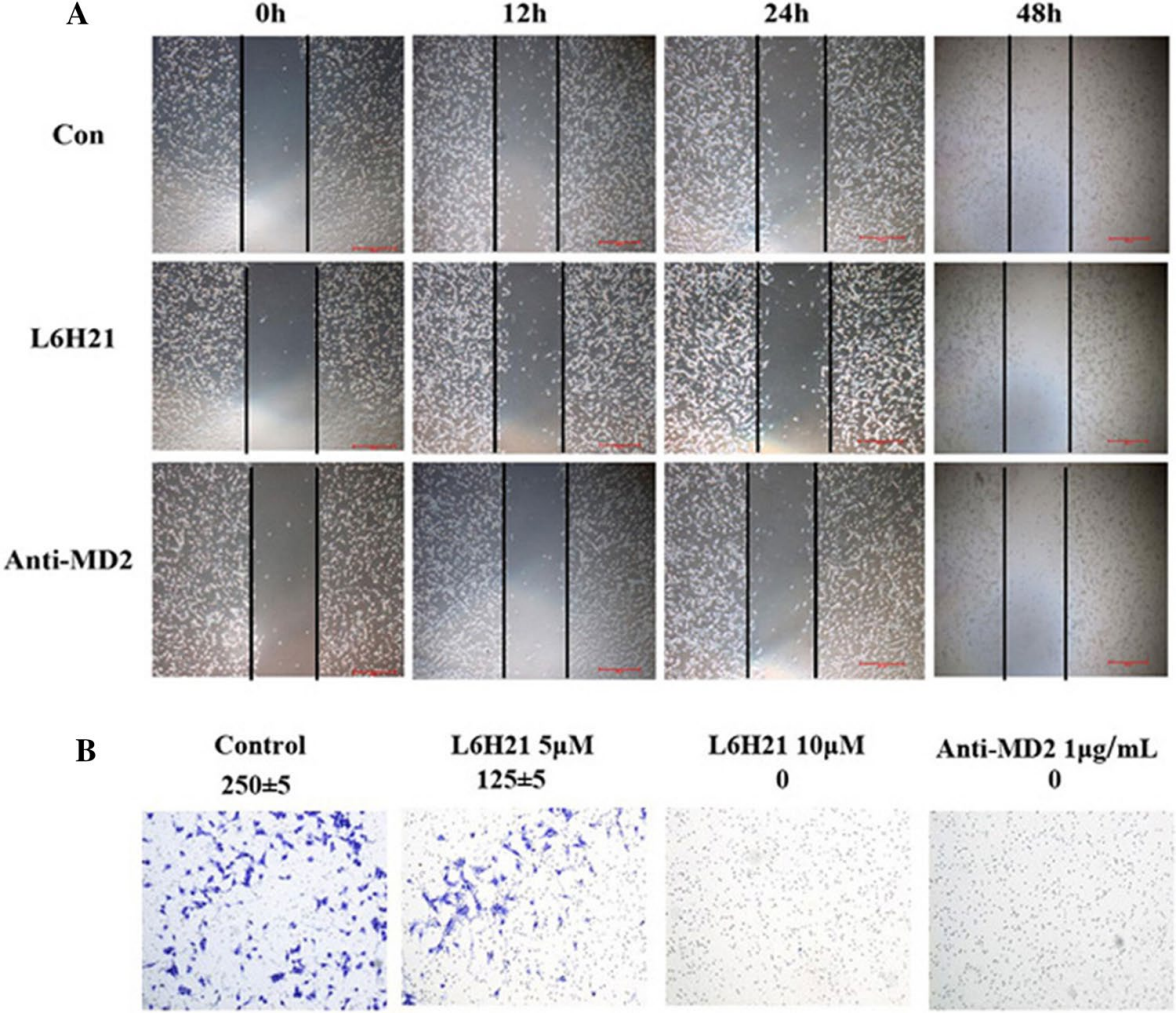
L6H21 inhibition of 4T1 cells migration and invasion. **a** L6H21 significantly inhibited the migration of 4T1 cells, The images (100 ×) were obtained by microscope. **b** L6H21 significantly inhibited the invasion of 4T1 cells (400 ×). In 5 μM L6H21 group, there were a few migrating cells observed compared to that of control group. While in 10 μM L6H21 group and in 1 μg ml^−1^ anti-MD2 group, there were almost no migrating cells observed

To study the effect of MD2 in cell migration, we adopted a scratch wound model in the presence of mitomycin C which inhibited proliferation. In these conditions, migration was significantly decreased in L6H21 group (10 μM) and anti-MD2 group (1 μg/ml) as compared to the control group. Next, we performed trans-well invasion assay to investigate the effect of L6H21 in 4T1 cell invasion further. As shown in (Fig. 3), there were significant differences of effect of invasion in control group, 5 μM L6H21 group and 10 μM L6H21 group. Moreover, there was almost no cell observed in 10 μM L6H21 group as well as in 1 μg ml^−1^ anti-MD2 group. These results indicate that L6H21 has a significant inhibition of migration and invasion in 4T1 cell concentration-dependently.

### L6H21 suppresses tumor progression in the nude mice

**Fig. 4:**
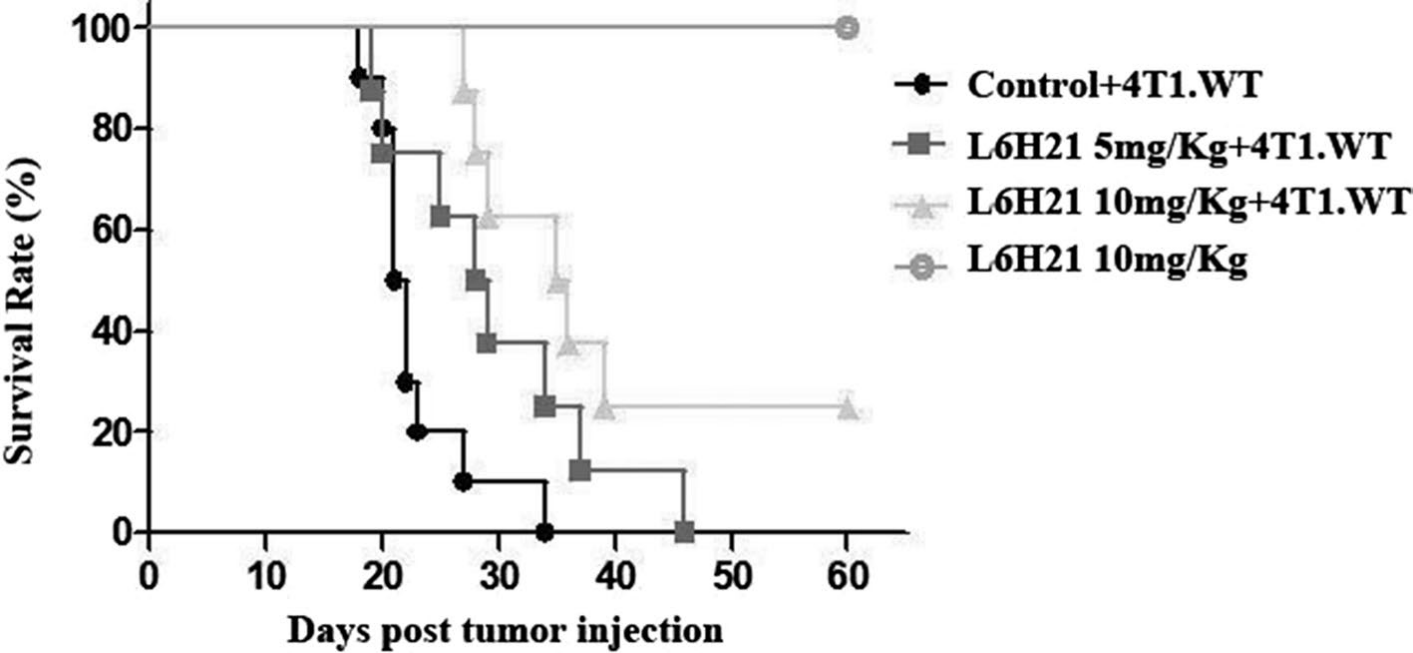
L6H21 enhanced survival in nude mice. Male BALB/c mice were randomly divided into four groups (*n* = 8 per group). 4T1-bearing mice were injected with 3 × 10^5^ 4T1 cells (i.v. through the tail vein) 3 days after being treated with L6H21 (at 10 mg kg^−1^ or 5 mg kg^−1^) and saline by intragastric administration, respectively, and the mice of the fourth group were only treated with L6H21 at 10 mg kg^−1^ but without inoculation of 4T1 cells. Each group of mice were then intragastrically administrated per day until death or day 60. The survival curve was made to analyse the survival rate

**Fig. 5:**
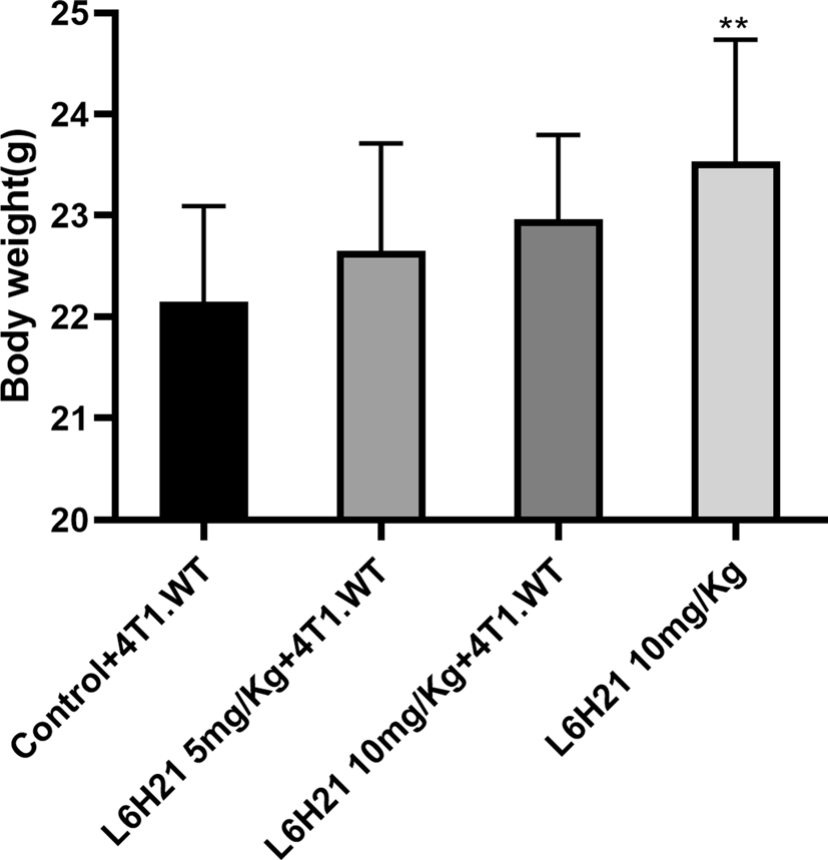
Body weight data after treatment. The body weight data after treatment were collected when mice were dead or at day 60. **p < 0.05 compared to the control group

To further investigate the role of MD2 in tumor progression, we assessed the effects of inhibition of MD2 on the progression of xenograft tumors in vivo. Figure 4 shows the survival rate of 4T1-bearing mice (treated with saline, 5 mg kg^−1^ and 10 mg kg^−1^ L6H21, respectively) and toxic control mice (L6H21 treated but without inoculation of 4T1 cells). As expected, the 5 mg kg^−1^ L6H21- or 10 mg kg^−1^ L6H21-treated mice survived significantly (*p* < 0.05) longer than the saline-treated mice. The mean survival times of 4T1-bearing mice treated with saline, 5 mg kg^−1^ and 10 mg kg^−1^ L6H21 were 23.8 ± 4.8, 30.9 ± 8.9, 40.4 ± 12.6 days, respectively. In addition, after treated for 60 days, there were still two mice (25%) survive in 4T1-bearing mice treated with 10 mg kg^−1^ L6H21. Interestingly, we also observed that there was none of the mice died from the toxic of 10 mg kg^−1^ L6H21 in 60 days. Figure 5 shows the body weight data of four groups after treatment. On the one hand, there were no differences in body weights in 5 mg kg^−1^ L6H21- or 10 mg kg^−1^ L6H21-treated 4T1-bearing mice as compared to the saline-treated 4T1-bearing mice (*p* > 0.05). But an upward trend of body weight could be seen as the concentration of L6H21 increase. On the other hand, the body weights of toxic control mice were significantly (*p* < 0.05) heavier than those of saline-treated 4T1-bearing mice. These data indicated that as an inhibitor of MD2, L6H21, could prolong survival efficiently with reliable security.

### Statistical analysis

For all data, statistical analysis was performed in SPSS 22.0 for Windows (SPSS Inc.). All data are shown as means ± SDs. The statistical significance between groups was obtained by Student’s *t* test or one-way ANOVA test and the significance level was set at *p* < 0.05.

## Discussion

Diverse studies have shown that TLR4 is associated with tumor development and progression. In breast cancer, TLR4 activation has been linked to both cancer inhibition and growth [10, 14–16]. Yang et al. reported that TLR4 expressed higher levels than any other TLRs and knockdown of TLR4 could actively inhibit proliferation and survival of human breast cancer cells MDA-MB-231. Functional analyses of ribonucleic acid interference (RNAi) against TLR4 revealed this successfully inhibited the growth and proliferation of MDA-MB-231 cells and resulted in a significant (*p* < 0.05) reduction of inflammatory cytokines [11]. In other work, 4T1 cells challenged with lipopolysaccharide induced tumor growth and metastasis, by increasing angiogenesis, vascular permeability, and tumor invasion [17, 18]. A total of 74 breast carcinomas were collected from patients to study the clinical relevance of TLRs in breast cancer. Tumors with high TLR4 expression, in mononuclear cells were found to have a higher probability of metastasis [19]. These studies suggest TLR4 involvement in breast cancer progression.

Although TLR4 plays an essential part in breast cancer progression, the role of its accessory protein, MD2, known for several years as an essential co-factor for TLR4 signaling, has not yet been clarified. One study highlighted that MD2 was overexpressed in highly invasive colorectal cancer cells (SW837), in poorly differentiated, moderately invasive colorectal cancer cells (HT-29), and in well-differentiated but non-invasive colorectal cancer cells (Caco-2) [17]. Another study reported that serum amyloid A 3, a major component of acute inflammation, binds to MD2 and activates the MyD88-dependent TLR4/MD2 pathway and thus facilitates lung metastasis [21]. Therefore, MD2 could be related to the degree of differentiation, proliferation, and migration capacity of cancers. However, there was few research focus on the relationship between the MD2 expression and the progression of breast cancer.

In the first stage of our study, we detected the expression of MD2 in several cell lines. As expected, the western blot assay showed MD2 is highly expressed in MDA-MB-231 s and 4T1 cells. Our preliminary results may probably indicate that MD2 is higher expressed in highly malignant cell lines than that in normal breast cells (Hs 578Bst) or MCF-7 cells (*p* < 0.05).

To investigate the role of MD2 in breast cancer progression, we next utilized L6H21, a new MD2 inhibitor, for down-regulating the expression of the MD2. And we performed experiments to observe the change of biological behavior of 4T1 cells using L6H21. MTT assay was performed and the results showed that L6H21 significantly inhibited the proliferation of 4T1 cells in dose-dependent manner, especially in 100 μM group. Moreover, scratch wound model and trans-well invasion assay demonstrated that L6H21 has a significant inhibition of migration and invasion in 4T1 cell. These results indicated that suppression of MD2 could effectively repress tumor cell proliferation, migration and invasion in vitro, in part consistent with the findings of Grondin et al., which was experimented with HT-29 cell [20]. To further determine whether MD2 regulates tumor progression in vivo, we used tumor xenografts by inoculating 4T1 cells in L6H21-treated or saline-treated nude mice. And we provided evidence that MD2 suppression by L6H21 prolonged survival in nude mice with hypotoxicity.

## Conclusion

In conclusion, our study indicates that 4T1 cells treated with L6H21 show progression inhibition in vitro. Moreover, L6H21 can significantly prolong survival in vivo. To our knowledge, this is the first report to describe the significance of MD2 expression to breast cancer cells in vitro and in vivo. Although the precise molecular mechanisms behind the altered expression of MD2 in breast cancer remain poorly understood, our data suggest that MD2 may be a promising candidate as a potential therapeutic target for breast cancer intervention.

## Data Availability

The dataset used during the present study is available from the corresponding author upon a reasonable request.

## Abbreviations

MD2: Myeloid differentiation 2
TLRs: Toll like receptors
PRRs: Pathogen recognition receptors
PAMPs: Pathogen-related molecular patterns
DAMPs: Damage-related molecular patterns
TRIF: TIR domain-containing adapter-inducing interferon-β
MYD88: Myeloid differentiation primary response
L6H21: (E)-2,3-dimethoxy-4′-methoxychalcone
FBS: Fetal bovine serum
OD: The optical density
LPS: Lipopolysaccharide
RNAi: Ribonucleic acid interference
